# Adherence to Sars-CoV2 vaccination in hematological patients

**DOI:** 10.3389/fimmu.2022.994311

**Published:** 2022-10-10

**Authors:** Justine Narinx, Margaux Houbiers, Laurence Seidel, Yves Beguin

**Affiliations:** ^1^ Department of Hematology, University Hospital Center (CHU) of Liège and University of Liège, Liege, Belgium; ^2^ Department of Information System Management, University Hospital Center (CHU) of Liège, Liege, Belgium

**Keywords:** vaccination, adherence, hematology, immunosuppressed patients, SARS-Cov-2

## Abstract

**Background:**

SARS-CoV2 vaccination efficiently prevents severe COVID-19, although hematological patients, particularly under therapy, respond less well. Besides vaccine efficacy, adherence to vaccination is essential for ensuring adequate protection of this vulnerable population.

**Methods:**

We evaluated the impact of a program aimed at maximizing patient adherence by comparing the rate of SARS-CoV2 vaccination of our hematological patients and a matched sample of the general population.

**Results:**

Vaccination rates were 88.9% among 2,156 patients, aged 65.2 ± 15.8 years (M ± SD, range 19-86 years). Rates differed considerably with age, i.e. 84.2% between 18-64 years and 92.4% above 65 years (p<0.0001), but not with sex. In the general population, rates were 76.3% overall, 73.0% between 18-64 and 86.7% above 65 years, all significantly lower than among patients, overall (Standardized Incidence ratio (SIR) 1.17; 95%CI 1.12-1.22, p<0.0001) as well as among younger (SIR 1.15; 1.07-1.24, p<0.0001) or older (SIR 1.06; 1.00-1.13, p=0.046) people. Vaccination rates increased to 92.2% overall (SIR 1.21; 1.16-1.27, p<0.0001), 88.5% in younger (SIR 1.21; 1.13-1.30, p<0.0001) and 94.8% in older (SIR 1.09; 1.03-1.12, p=0.0043) patients, after excluding those with medical contraindications, and further to 95.6% overall (SIR 1.26; 1.20-1.32, p<0.0001), 93.8% in younger (SIR 1.29; 1.20-1.38, p<0.0001) and 96.9% in older (SIR 1.11; 1.05-1.18, p=0.0004) patients, after excluding those not seen in hematology in 2021.

**Conclusions:**

Vaccination rates were significantly higher in hematological patients compared to the general population regardless of age, sex and municipality. Acceptance of Covid vaccines by hematological patients may be improved by targeted information campaigns carried out by trusted health care professionals.

## Introduction

In March 2020, the World Health Organization (WHO) declared coronavirus disease 2019 (COVID-19) caused by SARS-CoV2 (severe acute respiratory syndrome coronavirus 2) as a pandemic. Two years later, by March 2022, more than 469 million cases of infection have been diagnosed worldwide, resulting in more than 6 million deaths (1). Many efforts have been directed toward the development of vaccines against COVID-19 to avert further severe infections and deaths.

Immunosuppressed patients with hematological diseases are at higher risk of developing severe SARS-CoV-2 infections than the general population, with mortality rates exceeding 30% in some series (2). Patients with immunosuppression are also at high risk for prolonged infection, which may lead to a delay in the treatment of the hematologic disease with a risk of loss of disease control (3). SARS-CoV2 vaccination efficiently prevents severe COVID-19, although some hematological patients, particularly those under therapy, develop no or only partial post-vaccination immunization and therefore remain at risk of adverse outcome in the event of infection (4). Besides vaccine efficacy, adherence to vaccination is essential for ensuring adequate protection of this vulnerable population. A study conducted in December 2020 by The Leukemia & Lymphoma Society concerning attitudes and behaviors related to COVID-19 vaccination showed that a little less than one in five blood cancer patients reported vaccine hesitancy (5).

To improve the acceptance to vaccination, we decided to sensitize our hematologic patients by sending personalized letters or phone messages and by giving them the possibility to ask their questions to health care professionals. In this survey, we studied the adherence of the SARS-CoV2 vaccination in our hematologic patients and the impact of their sensitization by our different actions.

## Methods

**Figure 1:**
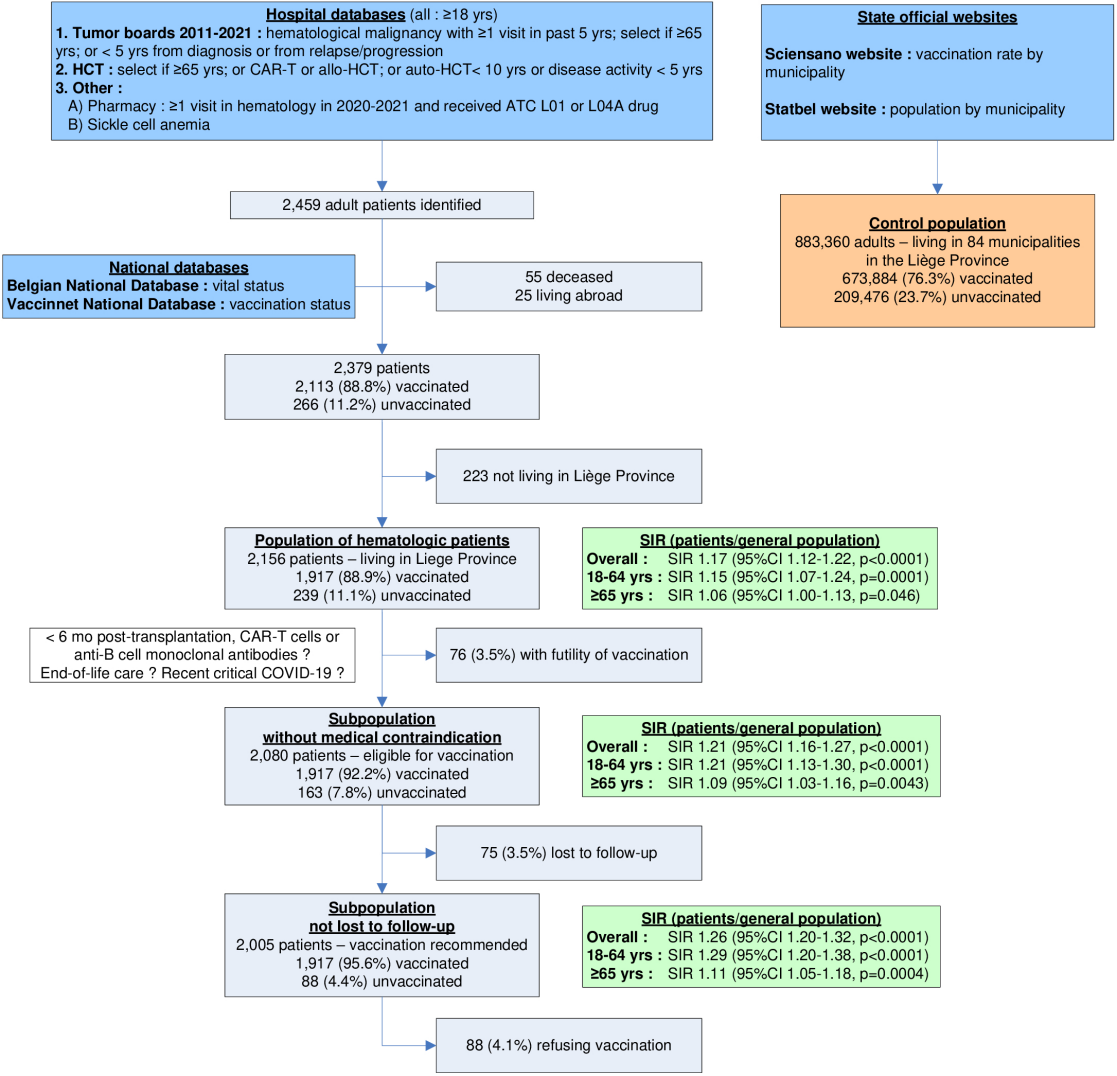
Consort diagram showing inclusion criteria for the population of adult hematologic patients (n=2,379). After removing patients not living in the Province of Liège, the overall patient population included 2156 patients. The sub-population of patients without medical contraindication (n=2080) and the subpopulation of patients not lost to follow-up (n=2005) were also analyzed. They were all compared to the adult general population of the Province of Liège (n=883,360). For each patient population, Standardized Incidence Ratios (SIR) with 95% confidence intervals were calculated for patients 18-64 yrs, ≥65 yrs and overall. SIR greater (smaller) than 1 indicate that vaccination rates in hematologic patients are higher (lower) than in the general population; 95% confidence intervals (CI) not encompassing 1 indicate that differences are statistically significant.

Preparing the national vaccination campaign in February 2021, we sought to identify immunosuppressed patients treated in the department of hematology at our tertiary academic hospital and eligible for priority SARS-CoV2 vaccination according to then applicable criteria in Belgium (**Figure 1**). We thus included adult (1) patients with hematologic malignancies diagnosed in 2011-2021 and having ≥1 visit in past 5 years if they were either ≥ 65 years of age or < 5 years from diagnosis or from relapse/progression (2); recipients of CAR-T cells or allogeneic transplantation; (3) recipients of autologous transplantation if ≥65 years of age or < 10 years from transplantation or with disease activity < 5 years; (4) hematological patients that received ATC L01 or L04A drug in past year; and (5) patients with sickle cell anemia We then conducted a program aimed at maximizing patient adherence by regularly sending personalized letters, contacting patients by phone and messages, discussing vaccination during hospital visits and providing easy access to health care professionals answering their questions. Newly diagnosed patients were included in the campaign between March and September 2021 when inclusions into our vaccination database were closed. We then checked the vital (Belgian National Database) and vaccination (Vaccinnet National Database) status of all patients at the cutoff date of September 17th, 2021, to identify those eligible for priority booster vaccination. Vaccination was considered completed after 2 doses of vaccine.

We aimed at evaluating the impact of this program by comparing adherence to SARS-CoV2 vaccination of our hematological patients and a matched sample of the general population. The study was approved by the Ethics Committee of the CHU of Liege under number 2022/25. We thoroughly reviewed the medical files of all unvaccinated patients to identify (1) medical conditions (< 6 months after hematopoietic transplantation, CAR-T cell therapy or anti-B cell monoclonal antibodies; end-of life care; recent critical COVID-19) rendering vaccination futile (2), last follow-up visit. We then classified them into medical contraindication, loss to FU (not seen in the hospital in 2021 and hence not exposed to the information campaign) or full eligibility.

On September 19th, we collected on the Sciensano website (6) the vaccination rates (available for individuals >18 and >65 years) in Belgium, the Liege Province and every municipality of the Province. We obtained the total number and proportions above 18 or 65 years of inhabitants in each municipality on the STATBEL website (7). Thereby, vaccination rates in patients were weighed according to the vaccination rate in their municipality (range 66-87%) to calculate the number of expected vaccinees in hematological patients, enabling the calculation of Standardized Incidence Ratios (SIR) with 95% confidence intervals. Results are considered significant at p<0.05. Calculations were done with SAS software, version 9.4.

## Results

By September 17^th^, 2021, 88.8% of the 2379 patients were vaccinated. Among the 2156 (90.6%) patients, aged 65.2 ± 15.8 years (M ± SD, range 19-86 years), living in the Province of Liege, 1917 (88.9%) were vaccinated (**Table 1**). Vaccination rates were similar in males (87.8%) and females (90.2%) (NS) but differed considerably with age, i.e. 84.2% between 18-64 years and 92.4% above 65 years (p<0.0001), ranging from 69.2% among those 18-30 years to 93.3% among those 90-100 years (**Table 1**). In the general population of the Province, the figures were 76.3% overall, 73.0% between 18-64 and 86.7% above 65 years. Vaccination rates were significantly higher among patients, overall (SIR 1.17; 95%CI 1.12-1.22, p<0.0001) as well as among younger (SIR 1.15; 1.07-1.24, p<0.0001) or older (SIR 1.06; 1.00-1.13, p=0.046) people (**Figure 1**).

**Table 1:** Vaccination rates in the population of 2156 hematologic patients living in the province of Liège, according to age, sex and municipality.

Variable	Categories	N	N vaccinated (%)	P value
**Total number of patients**		2156	1917 (88.9%)	-
**Age**	18-30 yrs	91	63 (69.2)	p<0.0001
	30-40 yrs	100	75 (75.0)	
	40-50 yrs	144	113 (78.5)	
	50-60 yrs	332	292 (88.0)	
	60-70 yrs	544	499 (91.7)	
	70-80 yrs	581	541 (93.1)	
	80-90 yrs	334	306 (91.6)	
	90-100 yrs	30	28 (93.3)	
**Age**	18-64 yrs	913	769 (84.2)	p<0.0001
	≥ 65 yrs	1243	1148 (92.4)	
**Sex**	Women	983	887 (90.2)	0.074
	Men	1173	1030 (87.8)	
**Municipality**	84 municipalities		(66-87%)	-

**Figure 2:**
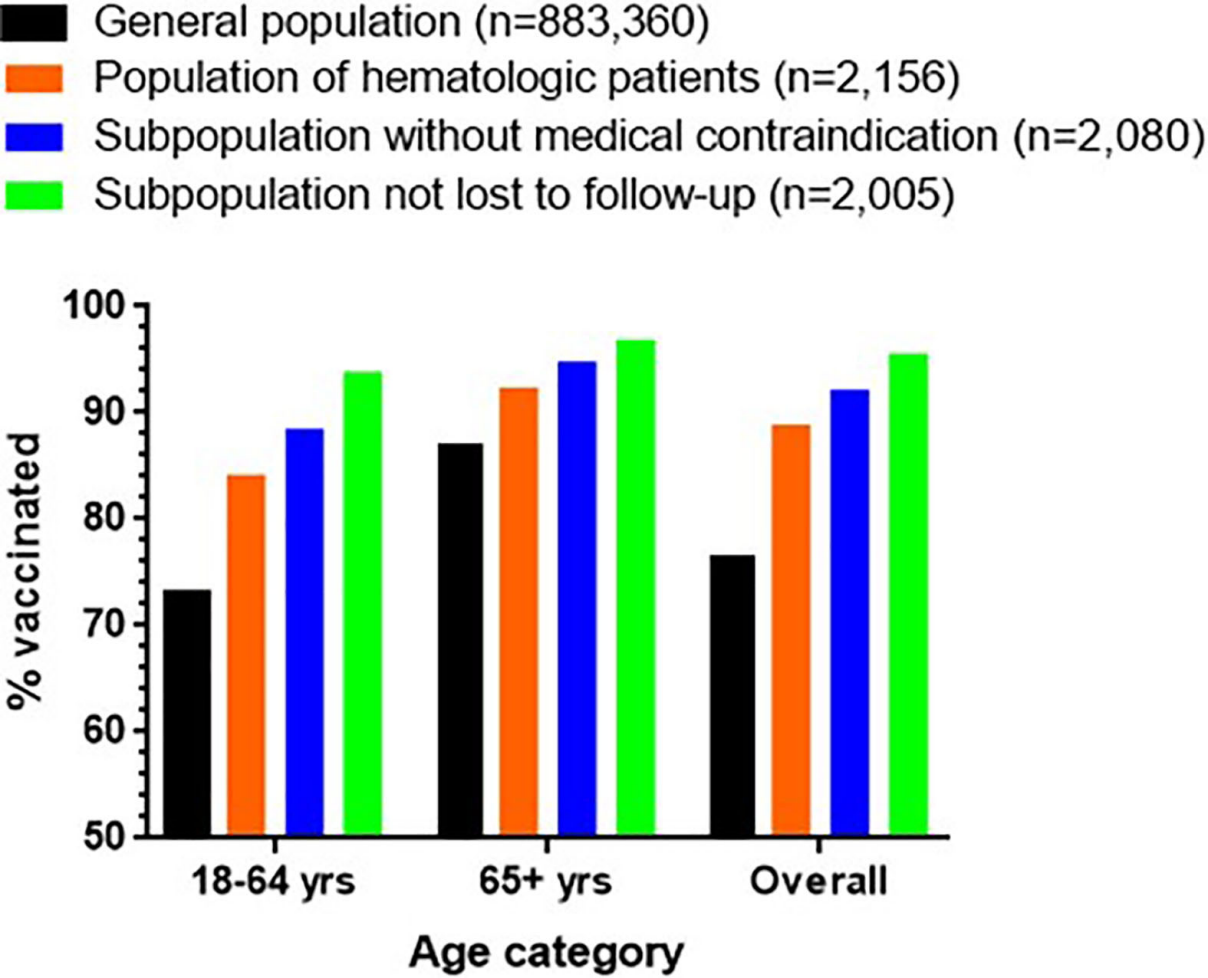
Vaccination rates in the general population as well as in the population of hematologic patients living in the Province of Liège. The sub-population of patients without medical contraindication (n=2080) and the subpopulation of patients not lost to follow-up (n=2005) are also displayed. The data are shown for patients 18-64 yrs, ≥65 yrs and overall.

For 76 patients (3.5%) the hematologist considered vaccination futile, 75 (3.5%) were lost to follow-up and only 88 (4.1%) refused recommended vaccination (**Figure 1**). Vaccination rates were 92.2% overall (SIR 1.21; 1.16-1.27, p<0.0001), 88.5% in younger (SIR 1.21; 1.13-1.30, p<0.0001) and 94.8% in older (SIR 1.09; 1.03-1.12, p=0.0043) patients when the 76 with medical contraindications were excluded (**Figure 2**). Excluding those without at least one visit in hematology in 2021, rates amounted to 95.6% overall (SIR 1.26; 1.20-1.32, p<0.0001), 93.8% in younger (SIR 1.29; 1.20-1.38, p<0.0001) and 96.9% in older (SIR 1.11; 1.05-1.18, p=0.0004) patients (**Figure 2**).

## Discussion

Whereas phase III studies demonstrated the safety and efficacy of various vaccines against severe Sars-CoV2 infection, their efficiency at the population level also depends strongly on adherence by the population. This is particularly critical in vulnerable patients, such as immunocompromised hematologic patients.

A review of 37 studies whose overall rate was fair identified that vaccine hesitancy was highest in persons who had a low perceived risk of getting infected or a low level of institutional trust, were not vaccinated against influenza, did not consider COVID-19 as severe, or strongly believed that vaccination was unsafe (8). A French survey measured hesitancy towards vaccination among 999 cancer patients at the end of 2020. Only 53.7% reported their intent to be vaccinated as soon as the vaccine becomes available, 29.7% considered they were not ready yet but likely to change their mind and 16.6% definitely refused vaccination (9). Later surveys of cancer patients conducted in 2021 also assessed patient attitudes towards vaccination and reported hesitancy or refusal rates ranging from only 6% refusal to 74% hesitancy/refusal at various periods and in various countries (9–22). Besides the large survey of patients with blood cancers already discussed (5), very few surveys specifically analyzed hematologic patients such as patients (n=147) with sickle cell anemia (23) or parents of children (n=113) undergoing hematopoietic transplantation (24). However, such surveys only consider patient attitude toward future vaccination, can be strongly biased by the type of patients answering among those solicited (some reporting response rates as low as 2%), sometimes include long-term survivors and provide no appropriate comparison with the general population. To our knowledge, no study reported actual vaccination rates among hematologic patients at the end of a full-fledged vaccination campaign and based on strong evidence instead of patient surveys.

In several surveys, patients reported that the opinion and advise of their oncologist would be determinant for the decision to be vaccinated (9, 10). One survey among 264 cancer patients reported a decrease from 29% to 17.5% hesitancy after a webinar organized by the hospital (22). Nevertheless, a survey conducted in Serbia in September 2021 showed that only 41.7% of patients with solid or blood cancer compared to 40% of the general population were vaccinated (25).

The Sars-CoV2 vaccination campaign in Belgium started in January 2021 with healthcare professionals and elderly people living in nursing homes. In March 2021, vaccination was extended to persons older than 65 years and patients at high risk because of medical reasons. Vaccination of younger and standard-risk persons started in June 2021 and was considered almost completed in September 2021 when the campaign for the third dose started. At that point, Belgium was one the countries with the highest vaccination rates among its general population. However, there was no procedure in place to identify high-risk people and vaccination in hospitals was abruptly stopped by the government after only 3 weeks in March 2021. Consequently, it was virtually impossible to prioritize patients on the basis of anything but age. Hence, vaccination timelines were quite parallel between our hematological patients and the general population, validating our comparison.

In our study, we aimed at improving patient information about Sars-CoV2 vaccination by sending personalized letters, contacting patients by phone and messages, discussing vaccination during hospital visits and providing easy access to health care professionals answering their questions. Vaccination rates were indeed significantly higher in hematologic patients (88.8% overall, 92.2% among those without medical contraindication, and 95.6% among those fully exposed to our information efforts) compared to the general population (76.3%) regardless of age, sex and municipality.

Adherence to Covid vaccines strongly depends on trust. Once trust is established between patients and the medical team, information campaigns such as ours can take root, disembodied messages gain strength, and acceptance of medically recommended but societally divisive protective measures that would otherwise be forgone or delayed may be improved. Thereby, immunosuppressed patients may better understand they are at higher risk of complications after SARS-CoV-2 infection and that vaccines confer safe and effective protection.

## Data availability statement

The raw data supporting the conclusions of this article will be made available by the authors, without undue reservation.

## Ethics statement

The studies involving human participants were reviewed and approved by Comité d’Ethique Hospitalo-Facultaire Universitaire de Liège. Written informed consent for participation was not required for this study in accordance with the national legislation and the institutional requirements.

## Author contributions

YB designed the study and interpreted the data. JN and MH collected and interpreted the data. LS performed the statistical analyses. JN wrote the first draft of the manuscript with contributions of YB and LS. All authors contributed to the article and approved the submitted version.

## Acknowledgments

The authors thank the physicians and nurses of the Department of Hematology for their care of the patients and their contribution to patient motivation towards vaccination.

## Conflict of interest

The authors declare that the research was conducted in the absence of any commercial or financial relationships that could be construed as a potential conflict of interest.

## Publisher’s note

All claims expressed in this article are solely those of the authors and do not necessarily represent those of their affiliated organizations, or those of the publisher, the editors and the reviewers. Any product that may be evaluated in this article, or claim that may be made by its manufacturer, is not guaranteed or endorsed by the publisher.

## References

[1] Coronavirus resource center home page . Available at: https://coronavirus.jhu.edu.

[2] Vijenthira A Gong IY Fox TA Booth S Cook G Fattizo B . Outcomes of patients with hematologic malignancies and COVID-19: A systematic review and meta-analysis of 3377 patients. Blood (2020) 136(25):2881–92. doi: 10.1182/blood.2020008824 PMC7746126 33113551

[3] Coray L Beyrer C Cohen M Michael N Bedford T Rolland M . SARS-CoV-2 variants in patients with immunosuppression. N Engl J Med (2021) 385:562–6. doi: 10.1056/NEJMsb2104756 PMC8494465 34347959

[4] Auletta J Boeckh M Dunbar C . American Society of hematology [Online]. General principles of COVID-19 vaccines for immunocompromised patients. version 3.0 (2022). Available at: https://www.hematology.org/covid-19/ash-astct-covid-19-and-vaccines.

[5] Rena C Jesper A Elisa W Sae-Hau M Lee M Gracia G . COVID-19 vaccine hesitancy among blood cancer patients. Leukemia and Lymphoma Society. Available at: https://www.lls.org/research/covid-19-vaccine-hesitancy-among-blood-cancer-patients.

[6] Sciensano . COVID-19 – bulletin épidémiologique du 19 septembre 2021 (2021). Available at: https://covid-19.sciensano.be/fr/covid-19-situation-epidemiologique.

[7] STATBEL . Population par lieu de résidence, nationalité (Belge/non-belge), état civil, âge et sexe (2021). Available at: https://bestat.statbel.fgov.be/bestat/crosstable.xhtml?view=c1649c18-ea66-4286-9310-2413e74134f8.

[8] Pires C . Global predictors of COVID-19 vaccine hesitancy: A systematic review. Vaccines (Basel) (2022) 10:1349. doi: 10.3390/vaccines10081349 36016237 PMC9415631

[9] Barrière J Gal J Hoch B Cassuto O Leysalle A Chamorey E . Acceptance of SARS-CoV-2 vaccination among French patients with cancer: a cross-sectional survey. Ann Oncol (2021) 32:673–4. doi: 10.1016/j.annonc.2021.01.066 PMC7846886 33529740

[10] Marijanović I Kraljević M Buhovac T Sokolović E . Acceptance of COVID-19 vaccination and its associated factors among cancer patients attending the oncology clinic of university clinical hospital mostar, Bosnia and Herzegovina: A cross-sectional study. Med Sci Monit (2021) 27:e932788. doi: 10.12659/MSM.932788 34772907 PMC8596742

[11] Di Noia V Renna D Barberi V Di Civita M Riva F Costantini G . The first report on coronavirus disease 2019 (COVID-19) vaccine refusal by patients with solid cancer in Italy: Early data from a single-institute survey. Eur J Cancer (2021) 153:260–4. doi: 10.1016/j.ejca.2021.05.006 PMC8149194 34183225

[12] Stoekle HC Sekkate S Angellier E Hervé C Beuzeboc P . Refusal of anti-coronavirus disease 2019 vaccination in cancer patients: Is there a difference between the sexes? Eur J Cancer (2021) 155:54–5. doi: 10.1016/j.ejca.2021.06.048 PMC8292033 34352570

[13] Peng X Gao P Wang Q Wu H Yan Y Xia Y . Prevalence and impact factors of COVID-19 vaccination hesitancy among breast cancer survivors: A multicenter cross-sectional study in China. Front Med (Lausanne) (2021) 8:741204. doi: 10.3389/fmed.2021.741204 34805207 PMC8595240

[14] Hong J Xu XW Yang J Zheng J Dai S Zhou J . Knowledge about, attitude and acceptance towards, and predictors of intention to receive the COVID-19 vaccine among cancer patients in Eastern China: A cross-sectional survey. J Integr Med (2022) 20:34–44. doi: 10.1016/j.joim.2021.10.004 34774463 PMC8559872

[15] Souan L Sughayer MA Abu Alhowr M . An update on the impact of SARS-CoV-2 pandemic public awareness on cancer patients’ COVID-19 vaccine compliance: Outcomes and recommendations. Front Public Health (2022) 10:923815. doi: 10.3389/fpubh.2022.923815 35937267 PMC9354075

[16] Day D Grech L Nguyen M Bain N Kwok A Harris S . Serious underlying medical conditions and COVID-19 vaccine hesitancy: A Large cross-sectional analysis from Australia. Vaccines (Basel) (2022) 10:851. doi: 10.3390/vaccines10060851 35746458 PMC9230066

[17] Tsai R Hervey J Hoffman K . COVID-19 vaccine hesitancy and acceptance among individuals with cancer, autoimmune diseases, or other serious comorbid conditions: Cross-sectional, Internet-based survey. JMIR Public Health Surveill (2022) 8:e29872. doi: 10.2196/29872 34709184 PMC8734610

[18] Overheu O Lendowski S Quast DR Marheinecke C Kourti E Lugnier C . Attitude towards and perception of individual safety after SARS-CoV-2 vaccination among German cancer patients. J Cancer Res Clin Oncol (2022), 1–8. doi: 10.1007/s00432-022-04099-7 35731276 PMC9215322

[19] Nguyen M Bain N Grech L Choi T Harris S Chau H . COVID-19 vaccination rates, intent, and hesitancy in patients with solid organ and blood cancers: A multicenter study. Asia Pac J Clin Oncol (2022). doi: 10.1111/ajco.13754 35043559

[20] Chun JY Kim SI Park EY Park SY Koh SJ Cha Y . Cancer patients’ willingness to take COVID-19 vaccination: A nationwide multicenter survey in Korea. Cancers (Basel) (2021) 13:3883. doi: 10.3390/cancers13153883 34359783 PMC8345425

[21] Moujaess E Zeid NB Samaha R Sawan J Kourie H Labaki C . Perceptions of the COVID-19 vaccine among patients with cancer: A single-institution survey. Future Oncol (2021) 17:4071–9. doi: 10.2217/fon-2021-0265 PMC8328088 34337969

[22] Kelkar AH Blake JA Cherabuddi K Cornett H McKee B Cogle C . Vaccine enthusiasm and hesitancy in cancer patients and the impact of a webinar. Healthcare (Basel) (2021) 9:351. doi: 10.3390/healthcare9030351 33808758 PMC8003419

[23] Jan H Waheeb A AlAhwal H Almohammadi A Al-Marzouki A Barefah A . Cureus. COVID-19 vaccine perception and hesitancy among patients with sickle cell disease in the Western region of Saudi Arabia. Cereus (2022) 14:e21026. 10.7759/cureus.21026 PMC8820497 35154996

[24] Skeens M Guttoo P Stanek JR Taylor K Stratz1 E Ardura MI . An exploration of COVID-19 impact and vaccine hesitancy in parents of pediatric hematopoietic stem cell transplant (HCT) recipients. Bone Marrow Transplant (2022) 57:547–53. doi: 10.1038/s41409-022-01587-9 PMC8785691 35075246

[25] Matovina Brko G Popovic M Jovic M Radic J Kladar M Nikolic I . COVID-19 vaccines and cancer patients: Acceptance, attitudes and safety. J BUON (2021) 26:2183–90. doi: 10.3390/vaccines10050672. 34761633

